# Errata: “Temporal Variability of Tungsten and Cobalt in Fallon, Nevada” & “A Retrospective Assessment of Occupational Exposure to Elemental Carbon in the U.S. Trucking Industry”

**DOI:** 10.1289/ehp.120-A57

**Published:** 2012-02-01

**Authors:** Paul R. Sheppard, Robert J. Speakman, Gary Ridenour, Mark L. Witten, Mary E. Davis, Jaime E. Hart, Francine Laden, Eric Garshick, Thomas J. Smith

**Affiliations:** 1 Laboratory of Tree-Ring Research, University of Arizona, Tucson, Arizona, USA; 2 Museum Conservation Institute, Smithsonian Institution, Suitland, Maryland, USA; 3 Internal Medicine, Fallon, Nevada, USA; 4 Department of Pediatrics, University of Arizona, Tucson, Arizona, USA; 5 Department of Urban and Environmental Policy and Planning, Tufts University, Medford, Massachusetts, USA; 6 Exposure, Epidemiology and Risk Program, Department of Environmental Health, Harvard School of Public Health, Boston, Massachusetts, USA; 7 Channing Laboratory, Brigham and Women’s Hospital and Harvard Medical School, Boston, Massachusetts, USA; 8 Department of Epidemiology, Harvard School of Public Health, Boston, Massachusetts, USA; 9 Pulmonary and Critical Care Medicine Section, Medical Service, VA Boston Healthcare System, West Roxbury, Massachusetts, USA

## Erratum

In the article “Temporal Variability of Tungsten and Cobalt in Fallon, Nevada” by Sheppard et al. [Environ Health Perspect 115:715–719 (2007)], “the Mann-Whitney test of medians” should have been “the Mann-Whitney test of differences in cumulative distribution functions.” This term was used in the last paragraph of the “Materials and Methods” and in Table 1.

## Erratum

Davis et al. have reported an error in their article “A Retrospective Assessment of Occupational Exposure to Elemental Carbon in the U.S. Trucking Industry” [Environ Health Perspect 119:997–1002 (2011)]. On p. 1001 of their article (the next to last paragraph of the “Discussion”), there was a coding error in the original calculation: The percentage of person-years prior to 1971 was 8% and not 1.1%, as stated in the article.

The authors apologize for the error.

This error has been corrected in the PDF version of this article.

